# Evidence supporting the safety of rapid refeeding protocols, using a food based approach, in a paediatric inpatient eating disorder program

**DOI:** 10.1186/2050-2974-1-S1-O17

**Published:** 2013-11-14

**Authors:** Shea Edsall, Terrill Bruere, Kellie Draffin, Amy Gregor

**Affiliations:** 1 Austin Hospital Melbourne, Australia

## 

Historically, a cautious approach to refeeding has been taken due to lack of evidence about refeeding syndrome. The aim of this study was to evaluate outcomes following the introduction of a more aggressive feeding protocol in a paediatric eating disorder program. This included commencing at a higher energy intake, more rapid energy increases and macronutrient manipulation of meal plans while maintaining a food based approach.

A restrospective audit of 38 Austin Hospital patients admitted for medical stabilisation of their eating disorder, using rapid refeeding protocols, were compared to previous refeeeding protocols. Thirty-seven patients (97%) were commenced on 8.2 megajoules (MJ) or more and increased to 11 MJ within one week. Previously, 23 patients (60%) had commenced on 6.8 MJ or less. With more intensive feeding 30 patients (79%) gained more than the target of 1 to 1.5 kilograms per week (compared to 35% previously). Evidence of refeeding syndrome was identified and treated in only two patients (5%). Only one patient required a lower energy intake (6.5MJ) with reduced contribution to energy from carbohydrates (40%) due to high risk of refeeding syndrome.

These findings suggest more rapid refeeding can be achieved safely with a food based approach in this patient group.

This abstract was presented in the **Care in Inpatient and Community Settings** stream of the 2013 ANZAED Conference.

